# Supplementary material for: Cerebral blood flow is associated with matrix metalloproteinase levels during the early symptomatic phase of concussion
Source: PLoS One. 2021 Nov 2;16(11):e0253134. doi: 10.1371/journal.pone.0253134 (PMC8562781; doi:10.1371/journal.pone.0253134)
Supplement: Attachment — Submitted filename: reviewer_response_1.5.docx [file pone.0253134.s001.docx]

**Overall response:** we would like to thank the reviewers for their helpful comments and suggestions. In addressing these points, we believe the clarity and scientific value of the manuscript to be significantly improved. Responses to individual reviewer points are provided below, and major changes to the manuscript text are highlighted in yellow.

**REVIEWER#1**

**Comment 1.1**: In a few areas, you mention "baseline" (line 212) levels or evaluations and "pre-injury" (line 178) values. It is unclear how these are measured given the description of how you enrolled your subjects.

**Response**: We apologize for the lack of clarity. All athletes participating in the varsity program had mandatory pre-season baseline clinical assessments which included administration of the SCAT. We have revised the Methods section to clarify this (p. 5, ln.107-110).

**Comment 1.2**: There are asterisks for Table 1, and it is unclear what these represent.

**Response**: This indicates post-concussion time points where symptom scores are significantly elevated relative to both controls and concussed athletes’ own baseline. We have amended the caption to state this.

**Comment 1.3**: The use of arterial spin labeling is interesting, but I believe the description in the methods section is very technical and is laborious for the reader. Perhaps using a description that is easier to decipher may help not to lose your audience.

**Response**: We have revised this section substantially to make the steps in ASL acquisition and processing clearer to a non-expert reader (p. 7-8, ln. 153-180).

**Comment 1.4**: There are several "hypotheses" listed in the introduction, and I believe that one should be focused on, using it to power the study according to a primary outcome, as you have both control and treatment groups--otherwise, this is an exploratory observational study. In other words, it was unclear to me what your actual objectives were a priori.

**Response**: Our main hypothesis is that among recently concussed individuals, those with higher MMP values will have lower global CBF values (i.e., a negative inter-subject correlation), indicating that MMP levels are related to cerebrovascular function. As a secondary hypothesis, it was predicted that the effect would be larger at early symptomatic injury, compared to both concussed athletes at RTP and uninjured controls, indicating that the effect is specific to the early phase of injury. We have revised the Introduction to focus on the main study hypothesis, as suggested (p. 4, ln. 87-91); we have also modified the Abstract (p. 2) and Discussion (p. 16, ln. 324-329; p. 17, ln. 344-345) to reinforce to the reader that this is the primary hypothesis.

**Comment 1.5**: line 77: I would not say that "human studies preclude. . ." as patients with severe TBI (not concussion, albeit) may have ventriculostomies and CSF sampling is possible for these biomarkers. More difficult, yes, but possible.

**Response**: This is an excellent point. We have amended the introduction to note that this is challenging, not impossible (p. 4, ln. 74-75). We have also added Discussion text (p. 20, ln.409-411) noting that this is a potential avenue for follow-up studies seeking to validate the present findings.

**Comment 1.6**: line 106: I believe the word "of" after "Imaging" could be omitted.

**Response**: This has been corrected.

**Comment 1.7**: line 107-108: I find that lack of concurrent imaging and blood samples a weakness as well as the timing of the blood sampling. You mention later in the discussion that "hyperacute" sampling showed higher levels of MMP, which I think is quite clinically relevant as usually concussed patients do not stay in the hospital and would just be seen in the ED and then leave.

**Response**: We agree that time of imaging and blood draws are important considerations. The present timeline is consistent with neuroimaging studies of sport-related concussion, allowing us to compare findings. As a study strength, the presence of significant correlations between MMPs and CBF indicates a relatively robust relationship that lasts beyond the acute window of injury. However, this may lead to under-estimation of effect sizes. Furthermore, generalization of these findings to the ER setting and early acute injury should be done with caution until replicated with data collected in this patient cohort. We have added these points to the discussion (p. 19, ln. 381-396).

**Comment 1.8**: Why did you choose the MMPs you chose? Also, did you consider utilizing other tight junction proteins such as occludin to confirm previously published findings. I believe that the timing of your sampling and perhaps the heterogeneity of the subjects (?) may have contributed to some findings that may lead to erroneous conclusions.

**Response**: The panel was selected, as it includes MMPs localized mainly to the vascular endothelium and previously identified as being relevant to vascular injury and TBI pathogenesis. This has been added into the Introduction text (p. 4, ln. 84-86). Regarding the potential investigation of other tight junction proteins, we now note in the Discussion that this will be an important next step in future work to better understand the mechanistic pathways (p. 19, ln.400-401). We have also added text discussing the limitations of our imaging timeline, and potential sources of demographic heterogeneity (p. 19, ln. 381-396). Although the present study found no significant impact of adjusting for age, sex and concussion history, there are likely other unmodeled sources of heterogeneity contributing to MMP and CBF variations, such as training load and recent subconcussive blows.

**REVIEWER#2**

**Comment 2.1**: In general the acute phase of TBI is considered to be 24h after the concussion, but maximum 3-4 days in more severe cases. Although literature varies on this aspect. Therefore I would discuss the matter how the classification of acute vs. non-acute TBI is assessed more in-depth. If in this discussion it is confirmed that the used median days of the analysis here (5 days) is not considered to be "acute", I would recommend to eliminate the word "acute" from the title and include this as a limitation of the study.

**Response**: Since clinical and biomarker literature adheres more consistently to the 24-48 hr definition of acute injury, and our median time post-injury is 5 days, we have changed the terminology to “symptomatic injury” throughout the manuscript to avoid any potential confusion. We have also amended the discussion to note that different MMP-CBF relationships may be identified in the acute 24hr post-injury window. This warrants further investigation, particularly if the goal is to translate the present findings into emergency care settings (p. 19, ln. 381-396).

**Comment 2.2**: I would find it necessary to show the CBF of brain regions separately and not merged, in which significant changes were found. To me it seems that in Figure 2B all regions where significant change in CBF was found, MMP and CBF values are merged to one figure. Although since part of the results and the discussion is based on the observation of spatial differences, the authors should really show the different brain regions separately, where significant change is supposed. This would increase the complexity of the paper and also would provide a more wider conclusion.

**Response**: We have conducted supplemental analyses examining whether taking the mean CBF value over all clusters yielded a good representative summary of cluster-specific associations between CBF and MMPs. For each of the contiguous clusters, we compared the cluster-specific regression coefficients to the all-clusters regression coefficient within a bootstrap resampling framework. None of the clusters deviated significantly at an FDR of 0.05, indicating that the overall mean was a good representation. One cluster did deviate at a nominal p<.05 uncorrected, and for completeness these results are reported in text. We have modified the Methods (p. 10, ln. 219-226) and Results (p. 14, ln. 291-297) to provide these details.

**Comment 2.3**: Please comment on why baseline "Symptom severity" and "Total Symptoms" are higher than RTP in Table 1.

**Response**: Although the difference is modest, this is consistent with previous publications in this cohort and prior reporting trends in the clinic. We have previously shown that this trend is correlated with improved mood state and reduced fatigue, likely due to a combination of anticipating return to play and being more physically rested at this time [Hutchison et al. (2017). JHTR, 32(3), E38-E48.] This has been noted in the Results text (p. 11, ln. 235-238).

**Comment 2.4**: Reference 4 and 6 are the same, or one of them is cited wrong, please correct.
**Response**: We have corrected the error.
