# Supplementary material for: Cerebral blood flow is associated with matrix metalloproteinase levels during the early symptomatic phase of concussion
Source: PLoS One. 2021 Nov 2;16(11):e0253134. doi: 10.1371/journal.pone.0253134 (PMC8562781; doi:10.1371/journal.pone.0253134)
Supplement: Attachment — Submitted filename: reviewer_response2_1.0.docx [file pone.0253134.s002.docx]

**Overall response**: we would again like to thank the reviewers for their detailed and helpful commentary, which we believe to have significantly improved the quality of the submitted manuscript. Specific responses to the remaining reviewer comments are provided below, and major changes in the manuscript text are highlighted in yellow.

**REVIEWER #1**

**Comment 1**: I just don't love Figure 2B and Figure 2C. Visually there does not appear to be good correlation. I also wonder if you think there is a possibility of ASL not being a good test to use in this setting? Is there a possibly limitation to the method by which you calculated CBF and could that be a reason there is not great correlation?

**Response**: Based on detailed investigation, the relationships plotted in Fig. 2B-C appear to be statistically valid. Rank-based spearman correlation with bootstrapped CIs found stable, moderately high correlations of approximately -0.63 for both MMP-2 and MMP3; this information has been added to the Results text (p. 14). Although there has been limited examination of associations between neuroimaging and serum biomarkers in concussion to date, these correlations are comparable in strength to the few extant ones (e.g., Kawata et al. (2020). Frontiers in neurology, 11; Marchi et al. (2013). PloS one, 8(3), e56805). In addition, regression diagnostics find no evidence of deviations from linearity or normality based on residual and QQ plots, nor evidence of high-leverage outliers based on the cook’s distance criterion (see figure R1 below). We have added Methods text noting that these tests were performed (p. 10).

**Figure R1**: diagnostic plots for (top row) MMP-2, and (bottom row) MMP-3 regression analyses.

Regarding the use of ASL to quantify CBF, it is a recognized protocol that has been validated against the “gold standard” of ^15^O-water PET. It shows good validity, with the added benefit that it uses arterial blood as an endogenous contrast agent and does not require any injections or exposing patients to ionizing radiation [see, e.g., Alsop, et al. (2015). Magnetic resonance in medicine, 73(1), 102-116.]. Although there are trade-offs with alternative CBF techniques (PET, SPECT, FNIRS) a detailed review is beyond the scope of this paper, particularly given the reviewer’s request to keep the discussion as concise as possible.

**Comment 2**: It was unclear to me how the two MRIs had by the subjects were used together to get the data. Did all subjects have two MRIs?

**Response**: We apologize for the lack of clarity. At a given imaging session, in addition to the ASL scan, all participants had a series of structural scans that served to characterize brain anatomy and rule out abnormalities that would complicate analysis or indicate more severe pathology. This included T1-weighted imaging to evaluate neuroanatomy, fluid attenuated inversion recovery (FLAIR) to assess for lesions and edema (appearing as hyperintensities) and susceptibility-weighted imaging (SWI) to check for signs of microhemorrhage (appearing as hypo-intense spots). This has been clarified in the Methods text (p. 7).

**Comment 3**: Paragraph 3 may not need to be in the introduction; this information may need to be only in the discussion or be omitted

**Response**: We have removed this paragraph from the Introduction text.

**Comment 4**: Lines 83-87 likely belong in the methods.

**Response**: We have moved this text to the appropriate Methods section (p. 5).

**Comment 5**: I know you improved the jargon with regard to ASL, but I believe it is only mentioned in the introduction now in the lines listed above, and not really explained prior to being mentioned.

**Response**: We have modified the Introduction to clarify what ASL is measuring (p. 4).

**Comment 6**: Having said that, the entirety of the methods if prone to technical jargon, and I understand that in some ways this cannot be avoided, but if possible, if you can simplify ANY of it, it will be a better read manuscript.

**Response**: We have made further minor edits to the Methods imaging section for clarity (p. 7-8), but we are somewhat limited by the need to provide sufficient detail about imaging and data processing that study findings can be replicated in future research.

**Comment 7**: Line 232: I recommend to remove "As anticipated" as this expresses conjecture, which usually is reserved for the discussion portion of the paper

**Response**: This line has been removed from the text

**Comment 8**: Line 234: similarly, I recommend removing "In contrast" simply because it is unnecessary.

**Response**: This line has also been removed from the text.

**Comment 9**: Lines 236-238 likely belong in the discussion as to "why?".

**Response**: This has been removed from the text, as it is not sufficiently important to justify expanding the discussion text.

**Comment 10**: Lines 249-250: there are points that belong in the discussion here

**Response**: This has been removed from the text, as it is not sufficiently important to justify expanding the discussion text.

**Comment 11**: Lines 287, 289: remove the word "moderate." It provides interpretation of results which belong in the discussion.

**Response**: This term has been removed from the Results text.

**Comment 12**: Finally, in general, the discussion is very long and wordy. I would try to limit it to 5 paragraphs (currently 8), and leave out anything not pertinent to discussing your findings, with the last two paragraphs reserved for limitations and conclusions.

**Response**: We have made significant efforts to reduce text in this section, by condensing and combining of the first 2 paragraphs (p. 16), and also by condensing and combining the 2 limitations paragraphs (p. 18), reducing the size of the discussion substantially.
